# A rare case of osteoblastoma combined with severe scoliosis deformity, coronal and sagittal imbalance

**DOI:** 10.1186/s12891-017-1902-9

**Published:** 2017-12-19

**Authors:** Lin-nan Wang, Bo-wen Hu, Lei Wang, Xi Yang, Li-min Liu, Yue-ming Song

**Affiliations:** Department of Orthopedic Surgery, West China Hospital, Sichuan University, No. 37 GuoXue Road, Chengdu, Sichuan 610041 China

**Keywords:** Osteoblastoma, Pain-provoked muscle spasm, Spinal imbalance, Rigid scoliosis, Early diagnosis and surgical excision

## Abstract

**Background:**

Osteoblastoma is a rare and benign tumor which requires early diagnosis and surgical excision. Scoliosis is a common presentation following osteoblastoma. It is considered due to pain-provoked muscle spasm on the side of the lesion. Few researches about osteoblastoma combined with severe scoliosis have been reported.

**Case presentation:**

A 14-year-old girl presents with progressive scoliosis deformity for 3 years, with gradually appeared low back pain and numbness of left leg. Radiographic results showed osteoblastic mass at the left side of L3-L4 with severe scoliosis deformity, pelvic obliquity and spinal imbalance. The patient underwent posterior tumor excision, spinal decompression, scoliosis correction, spinal fusion with auto-graft and instrumentation from T8-S1. The mass was found to be osteoblastoma. The patient had a full neurological recovery with no aggravate of scoliosis or spinal imbalance during the follow-up.

**Conclusions:**

This case emphasizes the importance of early diagnosis and surgical treatment of osteoblastoma. Early surgical excision will not only prevent neurological deficit but also the progression of scoliosis. Atypical scoliosis presence without pain requires carefully examination of whether a tumor exists.

## Background

Osteoblastoma is a rare and benign tumor characterized by increased osteoid tissue formation surrounded by vascular fibrous stroma and perilesional sclerosis. Spine location is observed in 36% of the tumor and mostly in the posterior elements [1]. It is usually combined with painful scoliosis due to pain-provoked muscle spasm on the side of the lesion [2]. The occurrence of scoliosis varies and rarely requires long segment fusion and scoliosis correction [3, 4]. To our knowledge, there are few osteoblastoma studies in which the subject also has severe scoliosis. Here, we describe a rare case of osteoblastoma combined with severe scoliosis deformity, coronal and sagittal imbalance.

## Case presentation

A 14-year-old girl was referred to our department whose chief complaint was progressive scoliosis deformity for 3 years, low back pain for 2 year and numbness in the left leg for 6 months. Her parents had initially noticed that her body leaned slightly to left while ambulating 3 years ago. No further attention was given until progression with low back pain. The local hospital diagnosed idiopathic scoliosis with a recommendation for brace treatment, but the torso imbalance developed along with left leg numbness.

Physical examination of the patient showed right-side spinal scoliosis with a razor back of 2.5 cm high when bending down. The patient could walk with no intermittent claudication. The right-side shoulder is 1 cm higher than the left-side. The left-side ilium is higher than the right-side, and the absolute length of the bilateral lower extremity is equal. A slight percussion pain was found on the left side of the waist. Hypoesthesia was found in the anterolateral thigh and extensor muscle strength of the knee that decreased to Grade 4. No hypoesthesia region or weaken myodynamia of other part was detected. The straight-leg raising test negative. No Babinski’s sign was noted bilaterally.

**Fig. 1:**
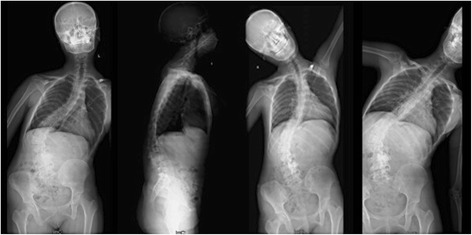
Posterior-anterior radiograph showed a 71 ° coronal plane deformity with an apex in the intervertebral space of L1 and L2. No significant reduction was found in side-bending image. The thoracic curve can be compensated with a Cobb’s angle of 43°. There is a 10.3 cm distant from the mid-point of superior border of sacrum to the C7 plumb line. This prompted severe coronal imbalance. Lateral radiograph showed sagittal imbalance (6.3 cm) and a 44° thoracolumbar kyphosis (T10-L2)

**Fig. 2:**
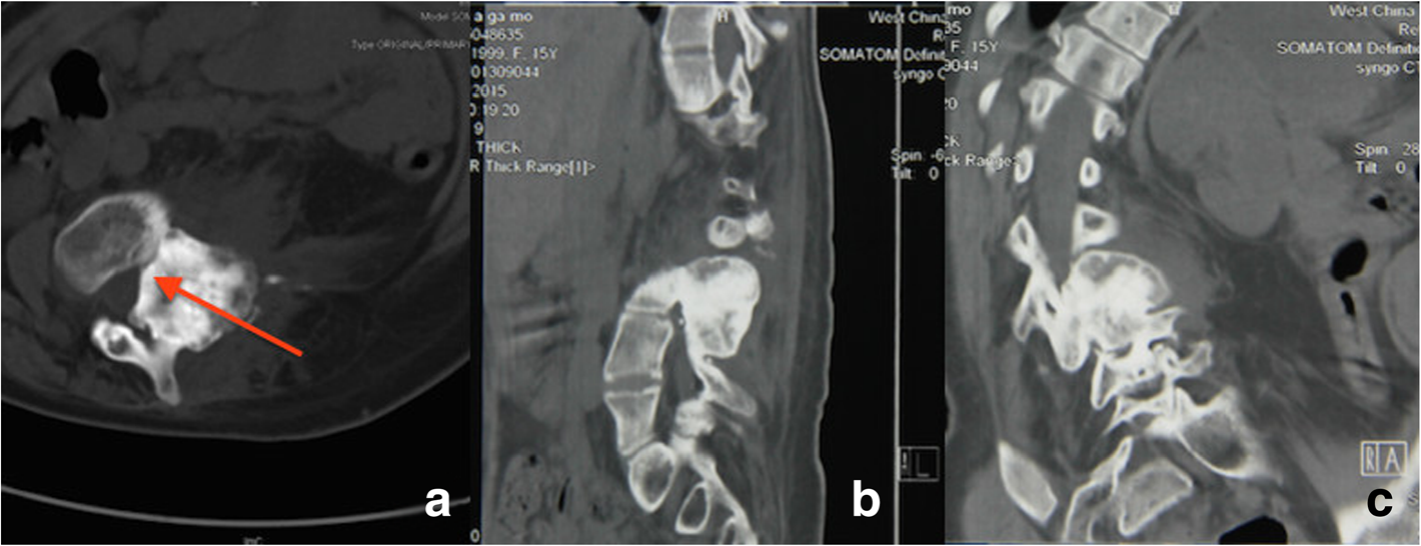
A three-dimensional computed tomography (CT) image of lumbar spine showed a 4*4*5 cm osteoblastic mass located on the left side of L3-L4, presented as a large expansile lesion involving the left pedicle with zygapophyseal joints and partial lamina. The mass had no obvious border to the normal bone tissue. Axial CT showed that the mass occupied the spinal canal and narrowed the nerve root canal of L3/4 (red arrow). **a** transaxial CT image, **b** sagittal CT image, **c** coronal CT image

**Fig. 3:**
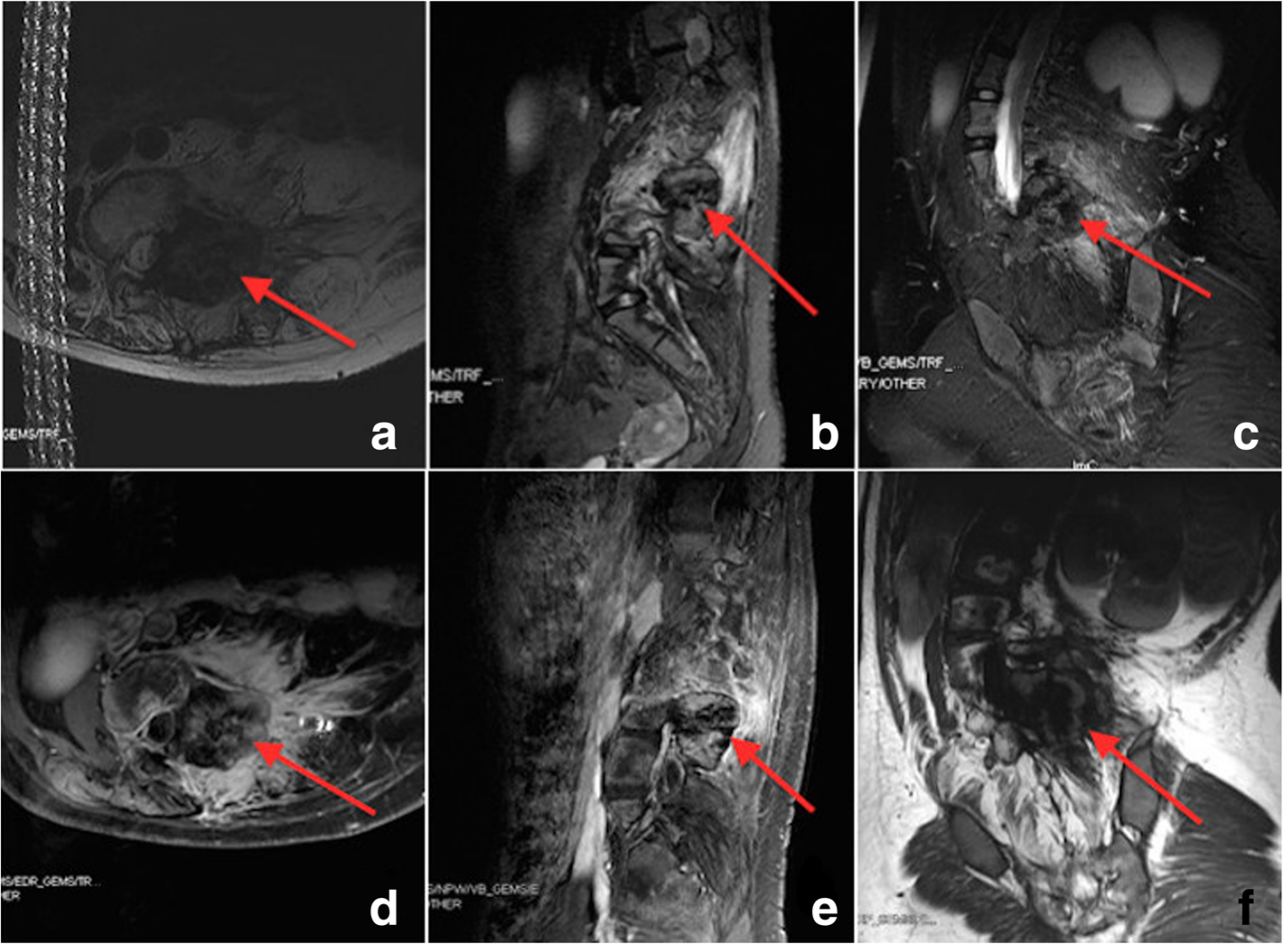
Contrast-enhanced magnetic resonance image of the lumbar spine demonstrated a heterogenic characteristic signal consisting of high and low signal intensities on both T1- and T2-weighted images (red arrow). Axial MRI showed narrowed spinal canal. (**a**, **d**) transaxial, (**b**, **e**) sagittal, (**c**) coronal T1-weighted images, (**f**) coronal T2-weighted images

The laboratory studies showed no increase in tumor markers and only a slight increase in B-ALP. Posterior-anterior radiograph showed a 71 ° coronal plane deformity with an apex in the intervertebral space of L1 and L2. No significant reduction was found in side-bending image. The thoracic curve can be compensated with a Cobb’s angle of 43°. There is a 10.3 cm distant from the mid-point of superior border of sacrum to the C7 plumb line. This prompted severe coronal imbalance. Lateral radiograph showed sagittal imbalance (6.3 cm) and a 44° thoracolumbar kyphosis (T10-L2) (Fig. 1). A three-dimensional Computed Tomography (CT) image of lumbar spine showed a 4*4*5 cm osteoblastic mass located on the left side of L3-L4, presented as a large expansile lesion involving the left pedicle with zygapophyseal joints and partial lamina. The mass had no obvious border to the normal bone tissue. Axial CT showed that the mass occupied the spinal canal and narrowed the nerve root canal of L3/4 (Fig. 2). Contrast-enhanced Magnetic Resonance Imaging (MRI) of the lumbar spine demonstrated a heterogenic characteristic signal consisting of high and low signal intensities on both T1- and T2-weighted images. Axial MRI showed a narrowed spinal canal (Fig. 3).

**Fig. 4:**
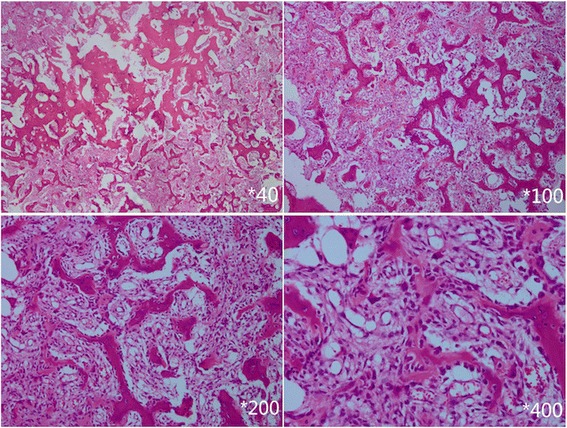
Pathological examination (hematoxylin-eosin staining) showed vascular soft-tissue component with significant areas of calcification and bone formation, resulted to be conventional osteoblastoma

The patient underwent posterior tumor total excision, spinal decompression, scoliosis correction, spinal fusion with auto-graft and instrumentation from T8-S1 using pedicle screw. Intraoperative exam showed that the tumor was located on the concave side of lumbar spine and narrowed the foramen of left L3-L4 with no obvious border with the normal bone tissue. Pathological findings suggested that the mass consisted of vascular soft-tissue component with significant areas of calcification and bone formation. This resulted in conventional osteoblastoma (Fig. 4).

**Fig. 5:**
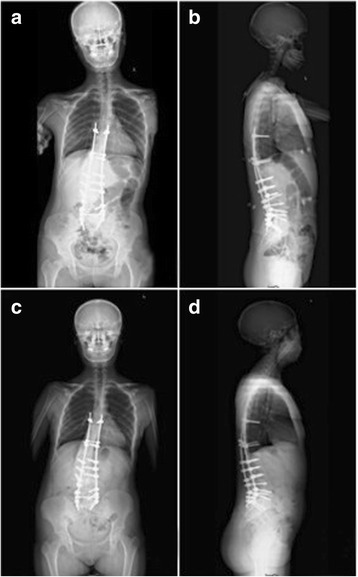
Postoperative anterioposterior radiographic image (**a**) showed Cobb’s angel decreased to 29 ° and distant from the mid-point of superior border of sacrum to the C7 plumb line reduced to 3.7 cm, indicating reconstruction of coronal balance. Later radiographic image (**b**) revealed the correction of thoracolumbar kyphosis and sagittal balance (1.9 cm). A 6-month follow-up radiographic image showed no aggravate of scoliosis or spinal imbalance (**c** and **d**)

Numbness of the left thigh was aggravated immediately after operation and relieved gradually within 1 week postoperatively. The patient could ambulate with brace support on the fifth postoperative day. Postoperative anterioposterior radiographic image showed Cobb’s angle decreased to 29 ° and distant from the mid-point of superior border of sacrum to the C7 plumb line reduced to 3.7 cm indicating reconstruction of coronal balance. Later radiographic image revealed the correction of thoracolumbar kyphosis and sagittal balance (1.9 cm). A 6-month follow-up radiographic image showed no aggravate of scoliosis or spinal imbalance (Fig. 5).

## Discussion and conclusions

Osteoblastoma was first proposed by Lichtenstein and Jaffe in 1956, which was then described as a category of osteoid- and bone-forming benign tumor other than osteoid osteoma [5, 6]. Of all osteoblastoma patients, 70% to 90% were younger than 30 years old [7]. 36% of osteoblastoma occurs in the spine particularly the posterior elements of the arch and spinous process [1]. Many studies about the incidence, natural history, character and treatment of osteoblastoma in the spine have been published [3, 8, 9].

Surgical resection is the most effective treatment for osteoblastoma in the spine [8–10]. In Boriani et al. research, they found that intralesional curettage was effective for early stage osteoblastoma (Enneking system stage II), but for more aggressive stage, intact resection had much less recurrence rate than non-intact resection [1]. Yin et al. compared total excision and subtotal excision in treating conventional osteoblastoma and aggressive osteoblastoma in 32 patients. Their results showed that total excision had a much lower relapse rate versus subtotal excision [8]. Li et al. reported that the most effective surgical option is en bloc resection of the tumor—especially for the aggressive osteoblastoma. Once the diagnosis is made, tumor excision should be performed as early as possible to prevent neurological injury [11]. In our opinion, intralesional curettage or subtotal resection can be effective and achieve satisfactory clinical outcome for conventional osteoblastoma. But for aggressive ones, total excision should be a better choice.

Scoliosis is a common presentation following osteoblastoma. Saifuddin’s literature review showed that 293 of 465 cases (63%) had scoliosis. They confirmed that asymmetric location of osteoblastoma in the thoracolumbar spine would be most likely to cause scoliosis [2]. Scoliosis in osteoblastoma patients is considered to be secondary to pain-provoked muscle spasm, but there are still cases of patients with scoliosis developing before pain occurred. Ozaki et al. reported 2 in 22 osteoblastoma patients revealed scoliosis before pain [3]. In this case, the patient presented initially with painless progressive scoliosis, which was diagnosed as Lenke V idiopathic scoliosis and suggested brace-treatment at local hospital. This delayed the detection of the mass.

To the best of our knowledge, early Lenke V idiopathic scoliosis may not present such severe torso imbalance and rigid scoliosis. We do not know if the tumor was present when scoliosis was diagnosed due to the lack of examination results from the local hospital. Todd et al. reported a case of thoracic spinal osteoblastoma occurring 9-years after idiopathic scoliosis correction surgery. However, tumor lesion of this patient is 4-segments higher than the apex [12]. In Saifuddin’s research, the apex of scoliosis is often the tumor lesion, but for lesion at lower lumbar spine, the apex is located above the lesion with pelvic obliquity [2]. As in our case, the tumor lesion is in the lower lumbar and apex located above the lesion with pelvic obliquity. This met Saifuddin’s metrics. Thus, rigid scoliosis, coronal and sagittal imbalance was much more likely due to the long-term tumor progression and fusion of adjacent bone tissue. The presence of severe and rigid scoliosis should be considered in choosing surgical options. We considered that tumor excision may not be sufficient for correction of scoliosis and torso imbalance for the long-term course and stiffness. Surgical options include tumor excision and scoliosis correction. Postoperative radiographic images showed satisfactory correction of scoliosis and imbalance; no progression was detected during follow-up.

This case emphasizes the importance of early diagnosis and surgical treatment of osteoblastoma. Early surgical excision will not only prevent neurological deficit but also the progression of scoliosis. Atypical scoliosis presence without pain requires carefully examination of whether a tumor exists.

## Abbreviations

CT: Computed tomography
MRI: Magnetic Resonance Imaging
